# Sleep in *Drosophila* and Its Context

**DOI:** 10.3389/fphys.2019.01167

**Published:** 2019-09-11

**Authors:** Esteban J. Beckwith, Alice S. French

**Affiliations:** Department of Life Sciences, Imperial College London, London, United Kingdom

**Keywords:** sleep, *Drosophila*, temperature, feeding, starvation, courtship, aggression, social interaction

## Abstract

A prominent idea emerging from the study of sleep is that this key behavioural state is regulated in a complex fashion by ecologically and physiologically relevant environmental factors. This concept implies that sleep, as a behaviour, is plastic and can be regulated by external agents and changes in internal state. *Drosophila melanogaster* constitutes a resourceful model system to study behaviour. In the year 2000, the utility of the fly to study sleep was realised, and has since extensively contributed to this exciting field. At the centre of this review, we will discuss studies showing that temperature, food availability/quality, and interactions with conspecifics can regulate sleep. Indeed the relationship can be reciprocal and sleep perturbation can also affect feeding and social interaction. In particular, different environmental temperatures as well as gradual changes in temperature regulate when, and how much flies sleep. Moreover, the satiation/starvation status of an individual dictates the balance between sleep and foraging. Nutritional composition of diet also has a direct impact on sleep amount and its fragmentation. Likewise, aggression between males, courtship, sexual arousal, mating, and interactions within large groups of animals has an acute and long-lasting effect on sleep amount and quality. Importantly, the genes and neuronal circuits that relay information about the external environment and internal state to sleep centres are starting to be elucidated in the fly and are the focus of this review. In conclusion, sleep, as with most behaviours, needs the full commitment of the individual, preventing participation in other vital activities. A vast array of behaviours that are modulated by external and internal factors compete with the need to sleep and thus have a significant role in regulating it.

## Introduction

Sleep is a behavioural state characterised by quiescence associated with a species-specific posture. This quiescence is quickly reversible to wakefulness, is accompanied by an increased arousal threshold compared to rest, and is homeostatically regulated, i.e., if removed it is compensated for. Historically, sleep has been an area of great interest and because of this, research on the subject is far reaching. Studies on many different species have contributed to this exciting field and, as a result, we are beginning to understand its function. This subject has been recently reviewed by Anafi et al. (2019). From ancient philosophy to modern technologies, no effort has been spared to study this enigmatic behaviour in numerous organisms both in the laboratory and in the wild (Rattenborg et al., 2017). The vast body of knowledge regarding sleep regulation has found a synthesis in the two-process model established by Borbély (1982). This simple and powerful paradigm describes that two processes, the circadian clock (process C) and the sleep homeostat (process S), work together to regulate sleep along the day. The former informs the oscillation of sleep pressure along the day while the latter conveys the need for sleep based upon duration and quality of previous wakefulness. If sleep is disrupted, the S process gains weight and is able to overcome the C process, pushing sleep into a period of the day that is normally associated with wakefulness. This compensatory sleep is a hallmark of the homeostat and is often referred to as “rebound sleep.” Although the Borbély (1982) model is still a standard in the field and an instrumental framework to study sleep, accumulating evidence that we review here suggests that sleep regulation goes beyond these two central processes.

Sleep is critical for fitness. In mammals, it is necessary to sustain physical and cognitive performance (Krause et al., 2017), and it actively supports the acquisition of long-term representations and synaptic homeostasis (Tononi and Cirelli, 2014; Feld and Born, 2017). It is also critical for development (Kayser and Biron, 2016) and immune function (Besedovsky et al., 2019). In insects, sleep also has an impact on fitness affecting reproductive output (Potdar et al., 2018), susceptibility to acute oxidative stress (Hill et al., 2018), and development (Kayser and Biron, 2016), and is important for learning and memory recall (Beyaert et al., 2012) and synaptic homeostasis (Gilestro et al., 2009; Bushey et al., 2011).

While there are clear fitness benefits inherent with sleep, at the same time, it can be a costly behavioural state. Being essentially offline means that animals are unable to engage in other essential activities, such as foraging or mating. This is evident in the strategies employed by different species in their attempts to balance their need for sleep and remain safe (Rattenborg et al., 1999; Voirin et al., 2014; Tisdale et al., 2018), fed (Willie et al., 2001; Keene et al., 2010), or reproductively successful (Lesku et al., 2012; Potdar et al., 2018).

**FIGURE 1:**
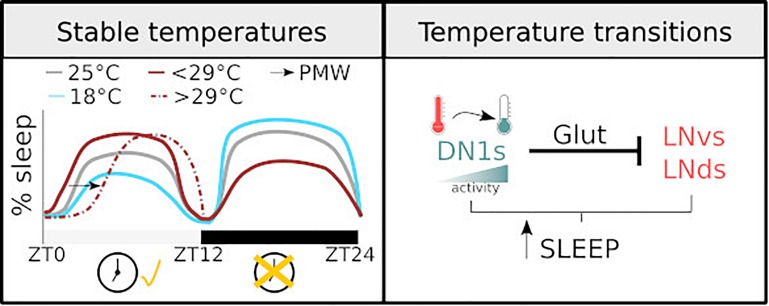
Diagram illustrating how temperature regulates sleep. **(Left)** The sleep profile of male flies under stable temperatures. The dashed line shows PMW observed with temperature >29°C. The changes observed at different temperatures are clock-dependent during the light phase and clock-independent during the dark phase. **(Right)** the mechanisms of sleep regulation in response to temperature change. The activity of DN1 neurons increases with temperature drops, resulting in an inhibition over the lateral neurons of the circadian clock and sleep promotion.

After key contributions to related fields such as courtship, aggression, circadian biology, and feeding, the value of *Drosophila melanogaster* as a model organism to study sleep was realised, and in the year 2000, the humble fruit fly made its debut in the sleep field (Hendricks et al., 2000; Shaw et al., 2000). Sleep is defined in *Drosophila* from a behavioural perspective: prolonged periods of immobility are used as a proxy for sleep. In particular, under the current shared operational definition, sleep is a period of immobility longer than 5 min, after which the flies exhibit a characteristic increase in arousal threshold. The definition of a sleep state in the fruit fly was first described through the use of video-recording or ultra-sound methods (Hendricks et al., 2000; Shaw et al., 2000). Later, the favoured tool for sleep analysis in *Drosophila* became the Drosophila Activity Monitor (DAM). This tool is still the most commonly used in the *Drosophila* sleep field. Activity is measured by counting each time a fly crosses the middle of the tube in which it is confined. Thus, sleep is scored when a period of 5 min or more occurs without a midline cross. Fruit flies, under laboratory conditions, show a characteristic rest-activity pattern where they are most active in anticipation of light to dark and dark to light transitions (Figure 1, left panel). Therefore, sleep occurs primarily during the middle of the day or night.

As described, sleep in *Drosophila* and other insects is largely measured through behavioural metrics, notably immobility. This is in contrast to the strategy employed to measure sleep in larger animals such as mice: Electroencephalography (EEG) is often used to determine when an animal transitions into a sleep state. Indeed, there is some ambiguity in using immobility as a definition of sleep in flies, yet the genetic tractability, fast life cycle, and low cost inherent in *Drosophila* research make it an important tool to understand many aspects of this behaviour and should be considered complementary to studies in other organisms. Early findings have demonstrated that *Drosophila* shares sleep characteristics comparable to many other species not limited to: clock control, homeostatic response to sleep deprivation, increase arousal threshold during sleep, species-specific sleep posture, and response to hypnotic/stimulant drugs (Hendricks et al., 2000; Shaw et al., 2000). These similarities together with plethora of genetic tools make it an appropriate model to study sleep (Helfrich-Forster, 2018).

Here, we review recent studies contributing to the idea that external conditions like temperature, social interaction, and food quality and availability, as well as resulting changes in internal state, such as levels of sexual arousal, aggression, hunger, or mating status, regulate sleep in adult *Drosophila* flies.

## Temperature Is a Regulator of Sleep

Pre-sleep behaviours of many species include nesting, huddling, and curling and is epitomised by the bedding behaviour in humans. Beyond the comfort associated with the initiation of sleep, these behaviours ensure body warming. In particular, skin warming is a sleep trigger in both humans and mice and is considered a sleep-permissive condition (Morairty et al., 1993; Raymann et al., 2008). This environmental factor induces sleep, via the median preoptic/medial preoptic hypothalamus (Harding et al., 2018; Komagata et al., 2019).

Being ectothermic, insects have a limited ability to thermo-regulate, thus, the interaction between sleep and temperature is fundamentally different to the one in mammals. However, insect development, metabolism, fecundity, as well as other physiological functions that determine fitness are, to a large extent, dictated by environmental temperature. There are two main categories of temperature sensation: the detection of an innocuous stimulus and the detection of painful temperatures (i.e., nociception). The perception of environmental temperature by adult flies relies on a family of temperature-regulated Transient Receptor Potential (TRP) channels tuned to different temperatures and are expressed in different cell types (Dillon et al., 2009). In particular, the dTRPA1 channel is critical for the detection of innocuous temperatures and instructs the distribution of wild-type flies along a thermal gradient ranging from 20 to 29°C (Hamada et al., 2008).

Temperature, as well as light, is a strong zeitgeber both in humans (Roenneberg and Merrow, 2007) and flies (Yoshii et al., 2016). As a result, fluctuations along the day constitute entrainment cues for the circadian organisation of rest-activity cycles in *Drosophila* (Maguire and Sehgal, 2015). Moreover, changes in temperature, either permanent or sudden, modulate sleep greatly. In particular, there are three contexts in which the effects of temperature over sleep have been studied: (1) the daily oscillations, (2) the seasonal variations between high and low temperatures, and (3) the abrupt variations that take place within a day.

In a seminal work, and before the documentation of a sleep state in flies, Majercak et al. (1999) described the distribution of locomotor activity, along a day, at different temperatures. Relative to 25°C,^1^ at high temperatures (29°C) flies increase their morning activity and delay their evening activity, thus becoming more active during the night. Meanwhile, at lower and stable temperatures (18°C), flies concentrate their activity during the light phase. Considering lack of activity as an index of sleep, we may take these results as the first description of sleep regulation under different, but steady, temperatures. Later works specifically measuring sleep have corroborated this schematic description: high temperature results in both an increased daytime sleep and a reduced nighttime sleep (Low et al., 2008; Ishimoto et al., 2012; Parisky et al., 2016).

Recently, complementary studies have focused on the acute response to temperature shifts (Parisky et al., 2016; Lamaze et al., 2017). In particular, changing ambient temperature from 22 to 29°C results in a reduction of sleep during the day and night. Temperatures higher that 29°C produce a clear increase in sleep latency at the beginning of the day. Lamaze et al. (2017) coined the term prolonged morning wakefulness (PMW) to describe this phenotype. Importantly, these observations support the original description. A parsimonious and encompassing interpretation would be that increases in temperature below 29°C result in increased sleep during the day and reduce sleep during the night. Higher temperatures, >29°C, result in an overall reduction in sleep, particularly at the beginning of the day, delaying it until the afternoon and the night. Considering that, given the choice, wild-type flies distribute at temperatures between 20 and 29°C (Hamada et al., 2008), a plausible explanation is that temperatures exceeding 29°C, in conjunction with the confined space associated with these types of behavioural experiments, may be triggering an escape response, manifested by a sustained reduction in sleep.

As a result of the described works, the field now has a detailed descriptive understanding of how sleep timing and amount in *Drosophila* is regulated by environmental change (Figure 1, left panel). This has opened the possibility to acquire a deeper mechanistic insight in the fascinating relationship between environmental factors and sleep.

Beyond variance in the extent of behavioural responses upon temperature change and some methodological differences, studies agree upon the idea that temperature dependent changes in sleep and activity during the light phase of the day are clock dependent. *period* (*per*) and *timeless* null mutants, i.e., flies with an impaired circadian clock, have an impaired behavioural response: these flies do not show an increase in daytime sleep after a temperature increase (Parisky et al., 2016). In addition, the posterior dorsal neurons 1 (DN1_p_s) cluster, which is part of the circadian network, is critical for temperature dependent sleep regulation (Guo et al., 2016). On the contrary, sleep changes during the night seem to be less dependent on a circadian regulation (Parisky et al., 2016).

One main caveat is that most studies investigating temperature dependent changes in sleep do not consider the natural correlation between light intensity/quality and temperature. On an average day, minimum temperature typically occurs just before sunrise. Subsequently, temperature rises reaching a peak sometime after solar noon, after which the temperature starts to drop. In addition, most experimental protocols change temperature very quickly at the onset of day, while in a natural environment this change does not occur so drastically. Thus, a heat shock together with the immediate initiation of the light phase is an artificial phenomenon that is unlikely to have driven the evolution of brain circuits controlling sleep. Notably, this concern is starting to be addressed: detailed studies have developed a more naturalistic approach to describe behaviour and the neuronal circuits controlling sleep in response to thermal changes (Currie et al., 2009; Yadlapalli et al., 2018). It is well documented that light and temperature are the strongest entrainment cues for the fly circadian clock. Employing a sophisticated paradigm Currie et al. (2009) showed that a 4°C oscillation is sufficient for effective entrainment. Moreover, they proved that the coupling between the external zeitgebers and the internal molecular clock allows the system to ignore thermal fluctuations that can be anecdotal signals,^2^ ensuring an appropriate entrainment (Currie et al., 2009). Furthermore, in a series of elegant experiments in which temperature and light were carefully administrated, Yadlapalli et al. (2018) demonstrated that DN1_p_s neurons are constantly monitoring temperature. This cluster responds to temperature drops with an increase in activity, inducing sleep. Interestingly, using GFP reconstitution across synaptic partners (GRASP) the same group showed that DN1_p_s contact two distinct clusters of the clock network, the small and ventral Lateral Neurons (sLNvs) and the dorsal Lateral Neurons (LNds). Upon activation, DN1_p_s inhibits, via a glutamatergic signal, the activity of these key clusters (Guo et al., 2016). The inhibition of the DN1_p_s by temperature increases and its impact on the sLNvs and/or the array of neurons that are downstream of this key cluster may explain, at least in part, the previously described PMW phenotype (Lamaze et al., 2017). See Figure 1 for graphical summary.

Finally, the regulation of the midday siesta by temperature and the adaptation to seasonally cold days are one of the best-studied examples of sleep regulation beyond the two-process model paradigm. At the molecular level, this regulation relies on thermo-sensitive alternative splicing in the clock gene *per*. In particular, the more frequent excision of the eighth intron (*dmpi8*) inhibits sleep on cold days because it generates a more stable version of *per* mRNA and protein that results in an earlier evening activity peak (Majercak et al., 2004; Chen et al., 2007). This event involves the serine/arginine (SR)-rich protein B52/SRp55, and the downregulation of this splicing factor in clock neurons reduces the efficiency of *dmpi8* excision. In addition, splicing efficiency of *dmpi8* affects transcript levels of the recently described gene *daywake* (*dyw*) (Yang and Edery, 2019). Cool temperature dependent splicing increases dyw mRNA which results in midday siesta suppression. This provides us with an elegant example of how sleep remains plastic in response to environmental changes (Zhang et al., 2018).

As a conclusion, sleep is highly affected by temperature, and thermoregulatory behaviours are in place from humans to flies. A comprehensive description of the behavioural adaptation of flies to different stable temperatures as well as the responses to gradual changes is now available. A more naturalistic approach to the interaction between light, temperature, and behaviour, together with the capabilities of *Drosophila* as a model system, will be key to elucidating the molecular and cellular basis of how environmental information is conveyed to sleep centres.

## Feeding and Sleep Are Mutually Exclusive Behaviours

Both sleep and feeding serve important functions, therefore allocation of time to each activity needs to be constantly assessed based on the animal’s level of tiredness and satiety state. Moreover, animals are particularly sensitive to nutritive changes in their environment and the resulting changes in internal state can influence sleep behaviour. Conversely, sleep deprivation can lead to changes in feeding behaviour (Koban et al., 2008): in humans sleep loss can lead to altered dietary choice, hyperphagia, and weight gain (Greer et al., 2013; Markwald et al., 2013). Understanding how sleep and appetite interact is a particularly relevant area of research and timely with emergence of public health crises such as obesity, which are symptomatic of modern lifestyles also associated with insufficient sleep.

*Drosophila melanogaster* feeds on fruits and microorganisms, such as yeast, associated with fruit. Beyond being generalist feeders, flies are sensitive to changes in their internal nutrient status. Consequently, this species has been extensively studied and used as a model to understand diet-related behavioural changes and the underlying mechanisms.

The circadian clock controls feeding both in mammals (Panda, 2016) and in flies (Murphy et al., 2016). *Drosophila* tend to increase feeding in the morning and have a minor peak in the evening which coincides with when they are most active (Xu et al., 2008; Murphy et al., 2016). In addition, feeding can promote sleep and hunger can suppress it. It is therefore not surprising, due to the mutual exclusivity of these two behaviours, that animals have neural networks that evaluate their needs and instruct behaviour accordingly. In this section, we will discuss the effect of starvation/satiation, dietary composition, and stimulants on sleep in flies as well as the neural networks and genes involved.

### Satiation Promotes Sleep

A period of quiescence following ingestion of a meal is observed in animals spanning many orders. For example, refeeding following starvation induces sleep in rats (Danguir et al., 1979), which appears to be dependent on cholecystokinin signalling (Shemyakin and Kapas, 2001). Likewise, *Caenorhabditis elegans* also shows an induction of quiescence after a high-quality meal, and this is dependent on insulin and TGF-beta (You et al., 2008). *Drosophila* also exhibit increased sleep immediately following a meal, typified by higher arousal thresholds, suggesting that sleep is deeper compared to pre-meal. Post-feeding sleep, also called postprandial sleep, positively correlates with volume ingested, and it is also dependent on dietary composition. Protein, salt, and to a lesser extent sucrose ingestion induce postprandial sleep (Murphy et al., 2016).

Induction of postprandial sleep is partly controlled by a group of Leucokinin receptor (Lkr) neurons, which arborise in the suboesophageal ganglion (SOG), in the lateral horn (LH) and in the fan-shaped body (FSB), areas of the brain known to regulate feeding, process olfactory information, and control sleep, respectively. Silencing of Lkr neurons reduces postprandial sleep specifically following protein feeding. However, Lkr silenced flies still exhibit postprandial sleep in response to ingestion of bulky, low nutrient food indicating that other pathways exist to induce sleep when volumetric information dictates. Yurgel et al. (2019) showed that disrupting the function of AMP-activated protein kinase (AMPK) in Lk neurons inhibited sleep in fed flies. This phenotype was specifically linked to an increase in activity in Lateral horn Lk (LHLK) neurons (Yurgel et al., 2019), suggesting that these may be downstream of Lkr neurons controlling post-prandial sleep.

Another key player in balancing the need to sleep and feed is a subset of Allatostatin A-positive (AstA) neurons allocated in the postero-lateral protocerebrum (PLP). When thermogenetically activated, these neurons promote sleep, reduce locomotion, and suppress feeding. Whether they are activated upon ingestion of food is not known but AstA neurons play a role in balancing these mutually exclusive behaviours and promote sleep at the expense of feeding (Chen et al., 2016).

### Starvation Suppresses Sleep

Starvation or caloric restriction is a consequence of food scarcity, which is a naturally occurring environmental stressor for many organisms. Animals encounter seasonal variations in food availability and competition for food sources, thus employ strategies to cope with and survive food deprivation. One strategy employed by animals is to increase activity, which is usually interpreted as an augmented effort to locate food. Upon short-term food deprivation, *C. elegans* exhibit increased foraging behaviour and heightened sensitivity to food-related chemosensory cues (Skora et al., 2018). Similarly, mice also exhibit more wakefulness and reduced sleep during starvation (Hua et al., 2018).

Not surprisingly, *Drosophila* also exhibit this behaviour. An early study by Connolly (1966) used a technique called “grid square” to manually count border crosses of starved and fed mixed sex groups of *Drosophila* on a perspex grid (Connolly, 1966). Using this method, the author observed that the food-deprived groups exhibited more border crosses than the fed groups and concluded that starvation induces locomotor activity. This is, to our knowledge, the first work describing an activity phenotype induced by starvation in *Drosophila*. Higher resolution and throughput techniques have since been developed allowing the reproduction of this phenotype, and a more detailed description of the behaviour.

We now know that in response to food deprivation fruit flies, as first discovered by Connolly (1966), increase their locomotor activity (both velocity and walked distance) (Lee and Park, 2004; Yang et al., 2015; Yu et al., 2016), suppress sleep (Keene et al., 2010), sensitise their gustatory and olfactory neurons to food-related cues (Root et al., 2011; Inagaki et al., 2014; Ko et al., 2015; Sonn et al., 2018), and more readily accept unpalatable foods (LeDue et al., 2016). Heightened sensory perception likely contributes to the increase in activity by fragmenting sleep and reducing arousal thresholds (Linford et al., 2012). These behavioural and physiological changes probably serve to increase the likelihood of finding and ingesting food in the vicinity but are sometimes considered counter intuitive: inactivity and sleep states are characterised by a lower metabolic rate and would be more consistent with energy conservation and longevity (Isabel et al., 2005; Stahl et al., 2017). Indeed, flies selected for starvation resistance through experimental evolution increase their sleep to conserve their energy stores (Masek et al., 2014). It is worth noting that after 48 h of starvation activity of wild-type flies does decrease (Bell et al., 1985); however, this latter behavioural alteration is likely a precursor to death and it is unlikely to be a reflection of adaptive behaviour.

Keene et al. (2010) described the starvation-induced sleep loss phenotype in detail. In a series of thorough experiments they demonstrated that food deprived flies began to exhibit sleep loss after 12 h regardless of whether starvation was initiated at the start of light period or dark period. During starvation, sleep became more fragmented and arousal thresholds were lower (Hasegawa et al., 2017). Following reintroduction of food, flies initially increased feeding and subsequently increased their sleep (Keene et al., 2010), which appears to be driven by postprandial mechanisms rather than as compensation for accrued sleep debt during starvation (Regalado et al., 2017).

Sleep suppression and food searching in starved conditions is initiated by the absence of food. Food scarcity is perceived in two main ways. Firstly, through the absence of gustatory stimuli and secondly, by internal nutrient sensing. The absence of food is communicated to brain regions that regulate sleep and locomotion via these two routes. We will now discuss the neural networks communicating external and internal nutrient deficit.

#### Perceived Absence of External Food Sources Suppresses Sleep

Lack of gustatory input is one indication that food is absent. Gustatory receptor neurons (GRNs) are housed in sensilla on the proboscis, tarsi, and wing margins. Different subsets are involved in detecting different foods such as sugars or amino acids and bitter or dangerous plant metabolites. GRNs project to the SOG where they synapse with projection neurons that report to higher brain centres such as the superior medial protocerebrum (SMP) (Talay et al., 2017). Considering that many sleep-related neurons arborise in the SMP (Aso et al., 2014; Donlea et al., 2018), it is likely to be an area where gustatory information is integrated into circuits governing sleep/wake behaviour.

Lack of GRN stimulation, particularly of those involved in the detection of nutritive and appetitive foods, plays a role in suppressing sleep and inducing locomotion during starvation. Two main lines of evidence support this view. First, feeding flies sweet but non-nutritive sugars such as arabinose (Yang et al., 2015), or low concentrations of D-glucose (Linford et al., 2015) which flies cannot survive on, does not trigger hyperactivity or suppress sleep (Hasegawa et al., 2017). However, flies with impaired sugar sensing do exhibit increased locomotion (Yang et al., 2015) and sleep suppression (Hasegawa et al., 2017) when fed arabinose. Second, activation of sweet GRNs using TRPA1 was also sufficient to induce sleep in starved flies (Linford et al., 2015; Hasegawa et al., 2017). Despite not playing a significant role in postprandial sleep induction in replete flies (Murphy et al., 2016), it is well evidenced that under starved conditions, sweet gustatory perception is sufficient to restore total sleep. It should be noted that gustatory receptors are also expressed in the gut as well as the proboscis, tarsi, and wing margins; thus, these receptors could play a role in internal as well as peripheral nutrient sensing (Park and Kwon, 2011). Hasegawa et al. (2017) showed that activation of cells expressing sweet gustatory receptors GR64a, GR43a, and GR5a using TRPA1 induce sleep under starved conditions. All of these genes are expressed in the proboscis and, with the exception of GR5a, are expressed in the gut. This suggests that peripheral detection is likely to play a more significant role in this phenotype.

Interestingly, repletion, which in theory could be conveyed through stretch reception in the oesophagus or the crop, does not seem to be a cue for restoration of fed behaviour after starvation. Instead, internal nutritive assessment of food ingested appears to shift behaviour from food searching to quiescent. This is evidenced through a finding that flies fed high concentrations (3M)^3^ of a tasteless but nutritional sugar called sorbitol exhibit activity levels that are equivalent to flies fed sugars that are both nutritional and sweet tasting (Yang et al., 2015). Further, it seems that flies ingesting nutritious sugars have less fragmented sleep and exhibit higher arousal thresholds compared to those that consume sweet non-nutritive ones (Hasegawa et al., 2017). These data suggest that sleep induction could be driven by taste whereas sleep depth and architecture maybe dependent on internal nutrient sensing.

#### Perceived Internal Nutrient Deficiency Suppresses Sleep

Recent work has elucidated how neural networks governing starvation-induced phenotypes may detect food scarcity through internal nutrient sensing. Insulin producing cells (IPCs), express *Drosophila* insulin-like peptides (DILPs) which have been implicated in starvation-induced sleep suppression: compared to a fed condition, DILP2 mRNA levels are reduced in the heads of starved flies (Cong et al., 2015). Interestingly, IPCs express Lkrs and receive input from LHLK-positive neurons. Knock-down of Lkr mRNA in IPCs eliminates starvation-induced sleep suppression. It is of particular importance that LHLK cells become active under starvation and their activity is dependent on levels of circulating glucose (Yurgel et al., 2019) as well as upregulation of the gene *translin* which is expressed in these cells (Murakami et al., 2016).

**FIGURE 2:**
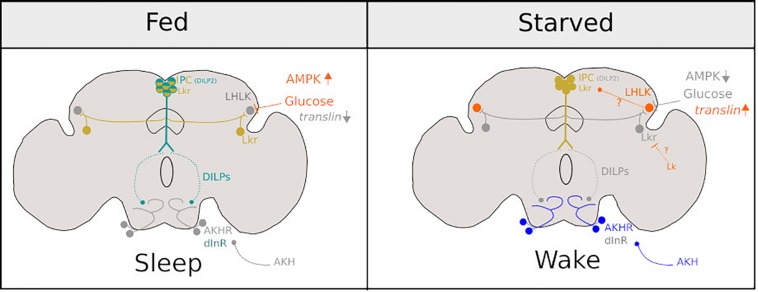
Diagram representing the neurons involved in promoting sleep under fed conditions **(Left)** and sleep suppression under starved conditions **(Right)**. Bright colours represent neuronal activity and grey represents neuronal inactivity under fed and starved conditions in *Drosophila.* Figure is adapted from Melnattur and Shaw (2019).

A key downstream target of DILPs is a pair of bilateral neurons in the SOG. In fed conditions, these neurons are inhibited by systemic signalling of DILPs and under starved conditions are activated by adipokinetic hormone (AKH) that results in increased walking (Lee and Park, 2004; Yu et al., 2016) and possibly suppresses sleep (Regalado et al., 2017). Thus, a model emerges, which is summarised in Figure 2. Under fed conditions, LHKR neurons are inhibited by circulating glucose. Under starvation, circulating glucose is reduced, and inhibition is released. This, in turn, suppresses the release of DILPS from IPCs perhaps through Lk signalling. Subsequently, DILP-dependent inhibition of circuits that promote hyperactivity (Yu et al., 2016) and may suppress sleep (Regalado et al., 2017) during starvation are alleviated. Interestingly, LHLK may also exert inhibition over Lkr neurons (distinct from IPCs) that have been implicated in the induction of postprandial sleep (Murphy et al., 2016). Murphy et al. (2016) do not address whether LHLK or other Lk-positive neurons (for example, those found in SOG) inhibit these Lkr neurons. However, it is possible that LHLK neurons have two downstream targets: the IPCs and Lkr neurons (those involved in postprandial sleep). Thus conceivably, LHLK neurons, whose activity is dependent on circulating glucose, could suppress or induce sleep depending on internally perceived satiety state via Lk signalling onto these two antagonistic circuits.

This circuitry may be the equivalent to pro-opiomelanocortin (POMC) and agouti-related protein (AgRP) neurons in the mouse hypothalamic articulate nucleus. AgRP neurons are activated in response to orexigenic hormones such as ghrelin, promote food searching behaviour, and suppress sleep under starved conditions. Conversely, POMC neurons are activated by insulin (which is a satiety signal) and promote sleep (Goldstein et al., 2018). Orexin neurons are also synonymous with feeding and sleep: they are sensitive to circulating glucose and ghrelin. Orexin neuron-ablated mice do not respond to food deprivation by increasing locomotor activity and sleep suppression (Yamanaka et al., 2003).

Dopaminergic neurons of the mushroom bodies (MBs) called DANs may also be part of the pathway(s) that evaluate and translate internal nutrient state into behavioural change in flies. MBs are involved in olfactory processing (Aso et al., 2014), learning [for review see Cognigni et al. (2018)], and sleep (Aso et al., 2014; Sitaraman et al., 2015b). Mushroom body output neurons (MBONs) are the main output of this brain region and are key regulators of sleep/wake behaviours (Sitaraman et al., 2015a). DANs can potentiate or depress MBON pre-synaptic zones and some have been shown to directly modulate activity of wake promoting MBONs, suppressing sleep upon activation (Sitaraman et al., 2015a, b). Interestingly, some DANs respond to food deprivation by increasing the size and density of their active zones. Inactivation of these same DANs impairs food seeking behaviour during starvation^4^ (Landayan et al., 2018; Tsao et al., 2018). DANs thus are sensitive to nutrient scarcity, and could regulate sleep and food searching under different nutritive conditions by modulating activity of wake promoting MBONs and/or by sensitising MBONs to food odours (Tsao et al., 2018) which may fragment sleep and reduce arousal thresholds. It should be noted that a *direct* link between starvation-induced potentiation of DANs and their subsequent involvement in sleep suppression/modulation of wake promoting MBONs cannot be made, but is highly congruent.

Another study has shown that paired anterior medial (PAM) neurons, a subset of DANs, become activated upon ingestion of sucrose but not by tarsal stimulation. Interestingly, activation by sucrose is more pronounced when flies are starved (Liu et al., 2012). Taken together it appears that DANs are modulated in response to both starvation and ingestion of high concentrations of sucrose (1 M), both of which can promote arousal and suppress sleep (Catterson et al., 2010; Murphy et al., 2016). Future work should investigate the link between starvation-induced potentiation of DANs and sleep suppression as it represents a highly promising avenue.

#### Genes Involved in Starvation-Induced Sleep Loss

New genes that regulate sleep during starvation are continually being discovered. Not surprisingly, a link between starvation-induced sleep suppression and metabolism is becoming evident. As discussed above, insulin is a signalling molecule involved in regulating sleep in response to food deprivation (a mechanistic summary is shown in Figure 2). Another major target of insulin signalling in insects is the fat body, analogous to the liver in mammals. This tissue is known to play an important role in regulating feeding and metabolism. A recent study has shown that fat body-specific knock-down of *phosphoribosylformylglycinamidine synthase* (*Ade2*), a gene that is highly conserved and involved in purine synthesis, reduces triglyceride levels and free glucose. These flies also exhibit lower levels of sleep, a finding that directly links fat body function, sugar/fat metabolism, and sleep (Yurgel et al., 2018). Partly in contention with this finding, several other studies have reported that the amount an individual sleeps is independent of body size and amount of lipid stored (Lee and Park, 2004; Meunier et al., 2007; Kent et al., 2009; Masek et al., 2014; Slocumb et al., 2015). These studies do however reach a consensus that the way in which animals mobilise energy stores and/or conserve them through modification of sleep/wake behaviour appears to translate into tolerance to starvation (measured through longevity). Epitomising this, Rover and Sitter flies possessing different alleles of the gene *foraging*, which codes for a cGMP dependent protein kinase G (PKG) (Sokolowski, 1980; Scheiner et al., 2004), differentially regulate metabolism and gene expression (Kent et al., 2009) under starvation. Compared to Rovers, Sitters have more carbohydrate stores, which they are able to mobilise under starved conditions and as a result exhibit less sleep suppression during food scarcity (Keene et al., 2010). Sitters are also more resistant to starvation measurable through increased survival on agar and an unencumbered ability to perform a learning and memory task (Donlea et al., 2012). Core differences in the way that the two variants respond to food deprivation metabolically may affect their perceived satiety level and actual nutritional requirements, which may explain the observed differences in their sleep levels.

Sonn et al. (2018) conducted the most comprehensive study looking at genes involved in starvation-induced sleep loss. Genes that are up-regulated in response to short-term starvation (6 h) are mostly involved in sensory perception, whereas genes up-regulated during chronic starvation (24 h) were concerned with metabolism or transmembrane transport of amino acids and metabolism of nucleotides. Most notably, following chronic starvation, serine levels were elevated together with three genes involved in serine biosynthesis, including *astray* (*aay*), a non-protein phosphatase part of the haloacid dehalogenase (HAD) family member that catalyses the last step in the biosynthesis of serine from carbohydrates. Flies harbouring a hypomorphic allele of *aay* failed to exhibit starvation-induced sleep suppression and had increased arousal thresholds (less waking in response to light pulse at ZT 18) (Sonn et al., 2018). Conversely, flies with a mutated *serine dehydrogenase* (*stdh*) gene, which codes a protein that breaks down serine, suppressed sleep under starved conditions to a greater extent than controls (Sonn et al., 2018). Importantly this study points to short- and long-term strategies that are in place to allow flies to cope with food deprivation. Following short-term food deprivation, sensory genes are upregulated. This may increase sensitivity of sensory neurons, making them more tuned to food-related cues. As flies become sensitised to external cues this may, in turn, optimise

food searching and may reduce arousal thresholds during sleep, ultimately increasing walking and reducing sleep.

Several genes expressed in clock cells also seem to play a permissive role rather than an instructive role in the starvation-induced sleep suppression phenotype. For example, inhibition over sleep promoting *Clk*-positive neurons is required for this phenotype (Keene et al., 2010). *cyc*^0^, *Clk*^jrk^, and *Clk*^ar^ mutant flies suppress sleep more than controls under starved conditions. Authors postulate that it is likely to be an antagonistic pathway overriding *clk-*expressing neuron input into sleep centres. These putative inhibitory neurons would become active during starvation. Additionally, neuropeptide F (NPF), which is expressed in l-LNvs and the FSB, among other brain regions, plays a role in exerting inhibition over sleep promotion (Hergarden et al., 2012). Loss of the NPF gene, which has been implicated in both feeding and sleep, eliminates starvation-induced sleep loss (Chung et al., 2017).

Taken together this suggests that genes regulating sleep in food deprived environments tend to encompass those involved in metabolism (Lee and Park, 2004; Meunier et al., 2007; Masek et al., 2014) and sensory acuity (Sonn et al., 2018).

Disruption to mechanisms that sense hunger state (and thus precede mobilisation of carbohydrate stores) appears to have knock-on effects on systems which prime the animals physiology and initiate food searching behaviour, both of which are required for endurance in starved conditions.

In summary, flies are equipped with mechanisms to sense internal (Murakami et al., 2016; Yurgel et al., 2019) and external (Linford et al., 2012, 2015; Yang et al., 2015; Hasegawa et al., 2017) nutrient availability to modulate sleep accordingly. Perturbation to this system ultimately results in aberrant feeding and sleep behaviour.

### Dietary Composition, Caloric Restriction, and Sleep

So far, we have discussed the effect of feeding and starvation on sleep. Next, we will discuss how dietary composition, amount of food, and its quality can alter sleep amount and its structure.

Concentration of dietary sucrose has been shown to affect sleep (Catterson et al., 2010). As discussed, starved flies exhibit hyperactivity and sleep loss, which is rescued by perception of a sweet gustatory stimulus (Linford et al., 2015; Hasegawa et al., 2017) such as sucrose at concentrations as low as 0.5, 1, and 5% (Yang et al., 2015). However, drastically increasing dietary sucrose from 5 to 35% reduces sleep and induces an intense locomotor activity in male and female flies (Catterson et al., 2010). Therefore, actual ingestion of very high calorie foods may counteract the sleep promoting effects of gustatory stimuli.

Dietary protein can also alter sleep: addition of 2% yeast to a 5% sucrose diet results in an increase of walking, compared to sugar alone diet, therefore, an altered sleep architecture, characterised by shorter bouts, and a concomitant reduction on the arousal threshold in males (Catterson et al., 2010).

Nutritive value of any given diet, for example carbohydrate:protein ratio, will impact sleep. Linford et al. (2012) compared sleep in male flies being fed 2.5% yeast mixed with two different concentrations of sugar: 2.5% (low sugar diet: LSD) or 30% (high sugar diet: HSD). Total amount of sleep was unchanged by diet, but on the LSD sleep became more fragmented, typified by shorter bout lengths. Further, flies on this diet were more easily aroused by light pulses, indicating lower arousal thresholds and less consolidated sleep. A plausible interpretation of these findings is that, in low nutrient environments, flies regulate their sleep architecture to detect and exploit food sources when they become available. Interestingly, flies on the LSD had less triglyceride stores than those on HSD but this did not appear to play a significant role in fragmenting sleep. Instead, gustatory perception of low sugar concentrations fragments sleep under nutrient scarce conditions since loss of sugar gustatory receptors rescued the fragmentation phenotype in flies fed a range of LSDs (Linford et al., 2012).

High sucrose alone and yeast mixed with low concentrations of sucrose promotes wakefulness and fragments sleep (Catterson et al., 2010), but in combination, yeast and high dietary sucrose consolidate sleep (Linford et al., 2012). This indicates that the quantity and relative proportion of protein to carbohydrate may have important phenotypic implications. Considering the importance of sweet gustatory perception on promoting sleep (Linford et al., 2012, 2015; Hasegawa et al., 2017), it is plausible that taste interactions and/or internal nutrient sensing may explain this apparent paradox (Linford et al., 2015).

An important consideration here is that dietary composition can also affect the gut microbiome (Sharon et al., 2010) thus prompting the question: are food-dependent sleep behaviours, in part, explained by changes in microbiota? Microorganisms living in the gut are known to assist with the breakdown of food and provide nutrients in their own right (Wong et al., 2014; Yamada et al., 2015), thus contributing to the nutritional and metabolic state of the animal. Microbiota also appear to interact with insulin signalling which is known to regulate feeding and sleep (Shin et al., 2011). While microbiome composition has been shown to affect mating choice (Sharon et al., 2010), egg laying, and feeding (Leitao-Goncalves et al., 2017), rather surprisingly, another study found that eliminating the microbiome in fruit flies has only very modest effects on sleep and locomotion compared to controls (Selkrig et al., 2018). Considering the wealth of literature describing the interconnection between the availability and quality of food on sleep, it is surprising that loss of gut microbiota has no effect on sleep (Selkrig et al., 2018). While admittedly we cannot definitive rule out whether microbiota composition has an effect on sleep; for now we can say with a degree of confidence that food-induced changes in sleep is mostly governed by taste and nutritional quality.

### Caffeine and Sleep

Plants produce secondary metabolites to protect themselves against pests that feed and/or lay eggs on their vegetal tissue. Thus, while foraging and selecting egg laying sites, fruit flies may have to contend with plant chemical defences. Caffeine is an alkaloid produced by many species, including coffee (*Coffea arabica*), tea (*Camellia sinensis*), and *yerba mate* (*Ilex paraguariensis*) making it one of the most widely consumed plant secondary metabolites by humans. Due to its psychostimulatory and wake promoting properties, its impact on sleep has been studied in numerous model organisms (Maximino et al., 2011; Panagiotou et al., 2018) including *Drosophila*. As in humans, caffeine has been reported to be an inhibitor of sleep in fruit flies (Andretic et al., 2008; Wu et al., 2009; Nall et al., 2016). In *Drosophila*, its effects are more prominent in females and during the night compared to day. While studies tend to focus on how caffeine psychostimulatory properties impact sleep (Andretic et al., 2008; Wu et al., 2009; Nall et al., 2016), it is likely caffeine also interacts with food intake and taste (Keebaugh et al., 2017), which, as discussed, have profound effects on sleep amount and partitioning. Caffeine tastes bitter to flies and reduces feeding by inhibition of sugar sensing GRNs and through activation of bitter-sensing neurons (Jeong et al., 2013).

Because caffeine interacts with sweet gustatory receptors (Jeong et al., 2013), and may do competitively, it is possible that elimination of caffeine-induced sleep loss by increasing the sugar in the food, a finding of Keebaugh et al. (2017), could be explained by this mechanism. To our knowledge, there is no convincing evidence showing flies that cannot taste caffeine still suppress sleep when fed it. GR93a is a gustatory receptor expressed in bitter-sensing neurons and is involved in caffeine detection (Lee et al., 2009). Flies mutant for this receptor do still exhibit caffeine-induced sleep loss, however, it is clear from this study that GR93a mutants still significantly reduce their intake of caffeine laced sucrose, probably because they still taste it. Importantly, many studies have demonstrated that other gustatory receptors are also involved in caffeine detection (Marella et al., 2006; Moon et al., 2006), suggesting loss of GR93a is probably not sufficient to eliminate peripheral detection. Interestingly, other bitter tasting molecules such as papaverine and quinine, which activate bitter-sensing neurons, and also inhibit sugar sensing ones, mimic the effects of caffeine on sleep yet do not have any known psychostimulatory effects (Keebaugh et al., 2017).

Other studies have shown that the neural pathways required for caffeine-induced sleep loss are in part shared with those putatively involved in starvation-induced sleep loss, namely the dopaminergic PAM cluster of the MBs. PAM-silenced flies slept the same amount on food laced with or without caffeine. Allowing flies to feed *ad libitum* on sucrose laced with caffeine over 24 h also activated these neurons (Nall et al., 2016). As discussed earlier, PAM neurons are potentiated by food deprivation and are involved in food seeking behaviours initiated by starvation (Landayan et al., 2018; Tsao et al., 2018). In addition, Liu et al. (2012) showed that PAM neurons do not respond to caffeine ingestion but do respond to sucrose ingestion (but not tarsal stimulation) in a manner enhanced by starvation.^5^ Thus, it is possible that the reduced feeding observed in flies presented with caffeine (Keebaugh et al., 2017) induces a hunger state which triggers food searching and sleep suppression. This may exacerbate the caffeine sleep phenotype. Another study has also implicated MB dopamine signalling in caffeine-induced sleep loss. Flies lacking dopamine receptor 1a (dDA1) are resistance to caffeine-induced sleep loss, a phenotype which can be rescued by expressing dDA1 in a subset of MB neurons driven by C747–GAL4 (Andretic et al., 2008).

While caffeine may influence sleep through its taste properties, there is certainly evidence to support that caffeine may also have psychostimulatory effects on sleep in flies. Firstly, some of the characteristics of caffeine-induced sleep loss and starvation are different. Caffeine affects night sleep more than day and it is more efficacious in females, whereas starvation reduces both day and night sleep in males and females. There is also evidence that the effects of caffeine and starvation are mechanistically separable (Murakami et al., 2016). It is interesting, however, that the pathways through which caffeine acts in fruit flies is not conserved in mammals (Yanik et al., 1987; Huang et al., 2005) or other vertebrates (Aho et al., 2017). The Seghal lab show that flies lacking the Adenosine receptor (dAdoR) gene, which is an important biological target of caffeine, have similar sleep levels to controls when put on caffeine laced food, but sleep bouts are slightly shorter. Instead of mediating effects through adenosine signalling, caffeine acts through cAMP/PKA pathways and antagonises PDE (Wu et al., 2009). In addition to the effects of caffeine on sleep, thus far it is unknown whether metabolites of caffeine contribute to the phenotype. Insects are equipped with cytochrome p450 enabling them to defend themselves against toxic plant secondary metabolites, such as caffeine, once ingested (Coelho et al., 2015). It is possible that caffeine is metabolised in the gut into compounds such as theobromine, paraxanthine, and theophylline, which could affect sleep via routes distinct from adenosine signalling.

In summary, animals encounter a range of nutritive landscapes and have to adapt their behaviour accordingly. Nutrient scarcity can drive food-searching behaviours, resulting in sleep loss. In addition, nutrient poor conditions can alter the architecture and depth of sleep making individuals more vigilant and easily woken. Ingestion of high calorie foods can also induce hyperactive behaviour and suppress sleep. Assessment of nutrient availability is largely achieved via peripheral detection by GRNs and through internal nutrient sensing. Both these systems can modulate sleep independently but likely work in synergy with one another. In addition to dealing with a variety of nutritive conditions, fruit flies have to contend with defence mechanisms employed by their food source to defend itself. Plant secondary metabolites can deter feeding and induce malaise or psychostimulatory effects upon ingestion, which can impact sleep. Food quality and availability therefore represents a major environmental factor that can alter the amount and structure of sleep.

## Social Interactions Have a Profound Effect on Sleep Regulation

Depending on their ecology, lifestyle, and social organisation, animals vary in the extent of their social interactions. Although light and temperature are the main synchronisers of the sleep-wake cycles, social cues and interactions can work as modulators of the circadian entrainment (Davidson and Menaker, 2003). For instance, blind humans who lack light entrainment can use social cues to adjust their circadian clock (Klerman et al., 1998). In addition, social jetlag can affect cognitive performance (Haraszti et al., 2014) and health (Roenneberg et al., 2012). The effect of social interactions on sleep and the circadian organisation of activity is observed across taxa and has been described in birds (Menaker and Eskin, 1966), fish (Kavaliers, 1980), and rodents (Crowley and Bovet, 1980; Tomotani et al., 2016). An outstanding work has shown that eusocial bees entrained using social cues inside of the hive can sustain long-lasting synchronisation that can overrule photic entrainment (Fuchikawa et al., 2016).

**FIGURE 3:**
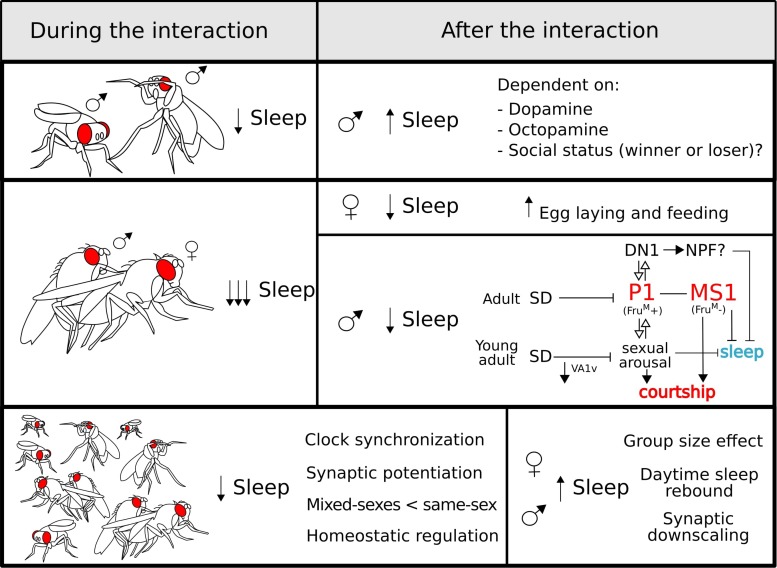
Sleep regulation during **(left panel)** and after **(right panel)** different social interactions: sexual **(top)**, aggressive **(middle)**, and group **(bottom)**. During all the interactions, sleep is reduced which is most poignant for sexual encounters. After copulation, males undergo a negative regulation of sleep controlled by sex-drive-related neurons (see the main text for a detailed explanation). For the group interaction **(bottom panel)**, some characteristics of this type of interaction are highlighted. After a social encounter, the effects on sleep regulation vary depending on the type of interaction and the sex of the fly.

Although *D. melanogaster* does not exhibit the complexity of eusocial insect colonies, these flies do engage in a repertoire of social interactions (Ramdya et al., 2017). There is solid evidence for the presence of social networks (Schneider et al., 2012) and collective behaviour in this species (Ramdya et al., 2015). Importantly, the most studied social behaviours in the fruit fly are simplified one-to-one interactions, namely, aggression (male–male encounters) and courtship (female–male encounters) for which the fly has emerge as a powerful workhorse to understand the neurogenetics behind these behaviours. Unfortunately, whether video recording or DAMs were used, sleep had mostly been studied at the individual level, with animals in isolation. Beyond the obvious advantage of measuring sleep in unperturbed and controlled conditions, this simplified and reductionist approach means that sleep is rarely studied in different social contexts, which are known to modulate sleep. Thankfully, in the last few years, efforts were devoted to address how social interactions affect sleep and vice versa in this powerful model system (see Figure 3 for a visual summary of this section).

### Male–Male Interactions

The increase in sleep after a stressful social situation seems to be a conserved feature of sleep regulation. In humans, sleep abnormalities have been reported in patients with posttraumatic stress disorder (Kobayashi et al., 2007), and insomnia is present in 80% of patients with depression (Armitage, 2007). Likewise, in mice, both acute (Fujii et al., 2019) or chronic (Henderson et al., 2017; Olini et al., 2017) socially induced stress produces an increase in sleep after the encounter.

Upon encountering another male, *Drosophila* males display agonistic behaviour that has proven to be an extremely useful model to study aggression (Kravitz and Fernandez, 2015). The confrontation results in the establishment of a hierarchy and represents a stressful situation, that reduces the amount of sleep (Gilestro et al., 2009; Beckwith et al., 2017; Machado et al., 2017) in a way that is dependent on the dimensions of the experimental arena (Lone et al., 2016). Notably, after the interaction, males show a clear increase in sleep that can take longer to manifest if the animals are housed at lower densities.

Consequently, the increased wakefulness triggered by the encounter with a conspecific, and the corresponding homeostatic regulation of sleep after it, constitutes an ecologically meaningful context to study sleep and its regulatory factors across species (Stahl and Keene, 2017). In *Drosophila*, beyond the clear homeostatic sleep recovery, further experiments would be needed to understand if the status of individuals after the fight (winner vs. loser) has a differential impact on sleep regulation or sleep need.

At the circuitry level, R2 neurons of the ellipsoid body (EB) act as a barometer for sleep pressure (Liu et al., 2016), and the activity of these neurons increases in response to both mechanical sleep deprivation and after a male–male encounter (Beckwith et al., 2017). Additionally, the change in sleep after a male–male interaction seems to be dependent on the dopaminergic system, while the circadian clock neurons are not necessary for this behavioural change (Lone et al., 2016).

Furthermore, the relationship between sleep and aggression in flies is bidirectional: acute sleep deprivation reversibly suppresses aggressive behaviours, competition for mating, as well as male courtship (Kayser et al., 2015; Chen et al., 2017; Machado et al., 2017). While a recent study has shown that long-term sleep loss has negligible effects on longevity in males (Geissmann et al., 2019a), the discussed data show that sleep is crucial for fitness in a social context.

Decisively, at the heart of the interaction between aggression and sleep lies octopamine. This neuromodulator is a well-described promoter of aggression (Hoyer et al., 2008) and suppressor of sleep (Crocker and Sehgal, 2008; Crocker et al., 2010). As expected, stimulation with an octopamine agonist is able to rescue the reduction in aggression (Kayser et al., 2015). However, despite ample evidence implicating octopamine in the regulation of aggression and sleep, the subsets of octopaminergic neurons involved in the interaction between these two critical behaviours have not been fully described.

### Female–Male Interactions

A female–male interaction is usually sexual in nature and involves courtship behaviour initiated by the male towards the female. The female will accept or reject the male’s advances in a manner dependent on its mating status. Courtship behaviour has been intensively studied in many species. Through the investigation of fly behaviour and neurogenetics, a detail description of the genes and circuits involved in this behaviour is available (Greenspan and Ferveur, 2000; Yamamoto and Koganezawa, 2013). This intense two-way interaction is crucial for perpetuation of the species and, in many contexts, it is prioritised over other behaviours including sleep.

#### Sexually Aroused Males Suppress Sleep

A clear example of how sexual arousal and the possibility of mating can regulate sleep was described in artic birds (Lesku et al., 2012). During the mating season, these polygynous birds suppress their sleep with no obvious sleep recovery at the end of the mating season. In addition, a key study shows that mating-related stimuli also suppress sleep in mice, a behaviour that is dependent on dopaminergic neurons from the ventral tegmental area (VTA) (Eban-Rothschild et al., 2016).

Interestingly, sleep suppression by the presence of a female or by the activation of the sexual arousal circuits was also reported in *Drosophila* (Fujii et al., 2007; Beckwith et al., 2017; Chen et al., 2017; Machado et al., 2017). Moreover, the presence of short-range non-volatile pheromones 7(Z),11(Z)-non-acosadiene (7,11-ND) and 7(Z),11(Z)-heptacosadiene (7,11-HD) can even suppress sleep rebound in mechanically sleep-deprived males (Beckwith et al., 2017). Shortly after pheromone exposure, males experience a series of physiological changes mediated by pheromone-sensing neurons located in the animals’ forelegs that express the *ppk23* receptor (Thistle et al., 2012; Gendron et al., 2014; Harvanek et al., 2017). These changes include the loss of triacylglyceride, an increased susceptibility to stress, faster aging and a significantly higher expression of NPF (Gendron et al., 2014). Importantly, NPF-expressing neurons are necessary and sufficient to drive this physiological switch. At the same time, these neurons represent a well-described arousal centre. NPF is a wake promoting NP and, as discussed, is a regulator of starvation-induced sleep loss (Chung et al., 2017). Thus, this NP represents a clear candidate to mediate the sleep reduction resulting from increased sex drive.

Beyond the data discussed above, the behavioural choice between engaging in courtship or sleep implies that courtship- and sleep-devoted circuits may interact to balance these competing drives. The expression of the male-specific splicing variant of the transcription factor *fruitless* (*fru*^M^) marks the neural circuits that govern male courtship. The P1 cluster of *fru*^M^-positive neurons in the protocerebrum integrates multisensory information and is a central hub for sex drive. P1 neurons indirectly activate the wake-promoting DH31-positive DN1 neurons (Chen et al., 2017) that are, in turn, part of the circadian network. Interestingly, this cluster is also *fru*^M^-positive and it is able to suppress sleep through DH31 secretion (Kunst et al., 2014). The interaction between these two clusters is bidirectional, showing a mutual activation that result in a positive feedback loop that biases behaviour towards courtship (Chen et al., 2017). Moreover, basal activity of the P1 neurons are negatively modulated by sleep deprivation, and, as mentioned, sleep-deprived males have reduced courtship behaviour (Kayser et al., 2015; Machado et al., 2017). Upstream of the P1 neurons lies a newly described cluster of octopaminergic neurons named Male-Specific 1 (MS1) (Machado et al., 2017). The activation of this cluster leads to a male-specific inhibition of sleep and promotes courtship in response to sex drive. The MS1 are *fru*^M^-negative, but there is a direct and sexually dimorphic synaptic contact with a group of *fru*^M^-positive neurons that innervate the SOG. Interestingly, the activation of MS1 neurons induces a broad activation of *fru*^M^-positive neurons, including the P1 cluster (Machado et al., 2017). Thus, the P1 neurons are crucial integrators of multiple sensory modalities and participate in the balance between courtship and sleep.

It is important to highlight that the interactions between networks that regulate these competing behaviours, and its hierarchical organisation, has not been fully elucidated. Regarding the link between the previously discussed networks and the reported sleep centres (Donlea, 2017), the constitutive activation of the sleep inducing neurons in the dorsal FSB can overcome sex-driven sleep inhibition (Machado et al., 2017). This finding reinforces the idea that the balance between sleep and courtship is bidirectional, i.e., it is not governed exclusively by sexual impulse. However, the exact interaction between the sex-drive clusters and this and/or other sleep centres like the R2 neurons of the EB is not clear. Untangling these connections will shed light on the complex balance between these two mutually exclusive behaviours.

Surprisingly, the female–male interaction and the concomitant reduction in *Drosophila* male sleep does not result in rebound sleep, a phenomena that is also observed in the artic polygynous birds study by Lesku et al. (2012). In flies, the experience of a prolonged sexual encounter, and probably the high levels of rejection that males experience due to the reluctance of females to re-mate, induces a strong reduction of sleep, which does not seem to be recovered (Beckwith et al., 2017). Further, the genetic activation of the P1 neurons or, to a lesser extent, the MS1 neurons, induces a reduction in total sleep that has a long lasting effect and does not result in a typical rebound sleep (Beckwith et al., 2017; Machado et al., 2017). A provocative idea could be that, in these conditions, sleep need is being recovered through a deeper sleep state; and these social interaction paradigms will be fundamental to address the relevance and the mechanisms underpinning such a state in flies.

These findings imply that a change in the internal state by sexual arousal can directly regulate sleep as well as modulate the homeostatic recovery of the lost sleep. Similarly, in humans, sleep quality and sleep onset are perceived to improve after achieving orgasm with a partner before bed (Lastella et al., 2019). It would be interesting to re-analyse the *Drosophila* data to address the immediate effect of mating in males and separate the effect of mating and the effect of courtship and rejection at the genetic and cellular level.

We mentioned that the interplay between sleep and courtship is bidirectional in flies, since acute sleep deprivation reduces sexual drive in a reversible manner. On a different time scale, sleep deprivation during critical periods of development has a long-lasting effect on adult behaviours like learning (Seugnet et al., 2011) and courtship (Kayser et al., 2014). In particular, the increased and deeper sleep phenotype observed in young individuals is required for the development of neural circuits necessary for courtship and mating. Consequently, sleep deprivation during the first 7 days of life specifically disrupts the development of antennal glomeruli to which *fru*M-positive neurons project. Male flies sleep deprived early in life have reduced VA1v glomerulus volume and have deficits in courtship behaviour (Kayser et al., 2014).

#### Mating Reduces Female Sleep

Regarding female behaviour, the effect of courtship on the regulation of sleep seems to be minimal. Two hours of exposure to a male does not result in a behavioural change in the courted but non-mated females (Geissmann et al., 2019a). However, mating triggers a series of changes in female behaviour. In addition to changes in egg laying, feeding, and courtship rejection, sleep shows a marked reduction after mating (Isaac et al., 2010; Zimmerman et al., 2012; Garbe et al., 2015, 2016; Geissmann et al., 2019a). Importantly, many of these changes, including a reduction in sleep, are reliant on the exchange of sex peptide from the male to the female during copulation (Isaac et al., 2010; Garbe et al., 2016). While it is generally agreed that copulation reduces sleep, the extent of sleep suppression varies between studies. While some reports describe a reduction of a 50% or less (Isaac et al., 2010; Zimmerman et al., 2012; Garbe et al., 2015, 2016), others reported a reduction close to the 90% (Geissmann et al., 2019a). We believe that these marked differences stem from two main reasons: method of sleep monitoring (DAMs vs. video tracking) and availability of protein-rich food. In brief, DAMs overestimate sleep and lack of protein inhibits egg laying, resulting in a gross underestimation of extent of behavioural change. Additionally, a finer description of behaviour after mating showed that sleep amount is not the only entity changed; instead, the entire behavioural profile is altered. Mated females walk less and spend a greater proportion of their time by the food performing micromovements, defined as a compendium of behaviours that includes feeding, egg laying, and grooming (Geissmann et al., 2019a).

This strong effect on sleep regulation is extremely informative and has implications for sleep research. A straightforward conclusion is that a good proportion of the sleep exhibited by virgin females can be exchanged for other physiological necessities and ecologically relevant activities. However, not all the measurable sleep disappears. Because of this, two scenarios arise: (A) sleep is exchanged for feeding and/or egg laying produces a sleep deficit in the mated female that can negatively affect its physiology or (B) virgin females show two components of sleep, one necessary and other one that can be exchanged at no cost. The fact that different reports fail to show sleep recovery after the behavioural switch would favour the second scenario. However, a component of the measurable sleep displayed by mated females confers a fitness benefit since sleep deprived mated females have reduced fecundity (Potdar et al., 2018). We believe that studying the behavioural switch after mating and the characteristics of sleep in mated females, which is likely the default state in wild fruit flies (Giardina et al., 2017), may be critical to understanding the regulation and functions of sleep.

### Group Interactions

Beyond the one-to-one interactions described above, socialisation in large groups also has an effect on sleep. This regulation of sleep is not restricted to the fly and is well documented in many insect (Eban-Rothschild and Bloch, 2012). For instance, in bees, a 2-day exposure to the colony environment generates an increase in sleep compared to the sleep shown by isolated bees of the same age and cast (Eban-Rothschild and Bloch, 2015).

In nature, *D. melanogaster* flies are found around food and oviposition sites (Shorrocks, 1972) forming mixed-sex groups that show evidence of social networks and collective behaviour (Ramdya et al., 2017). Hence, studying sleep regulation by social interaction within complex groups comprised of a genetically tractable organism, in a natural environment, may lead to meaningful observations. At the same time, it represents a methodological challenge since there are many different variables, which can influence the nature of any given interaction (e.g., sex ratio, density, food, and space availability). Likewise, the sleep of one or many individual flies within a group of interacting flies needs to be assessed.

In an elegant set of experiments Levine et al. (2002) showed that chemosensory cues involved in social communication are strong regulators of the rest-activity rhythms. They evaluated the locomotor rhythms (which we take as a proxy for sleep) of individual male flies following social isolation or group housing. Flies previously housed in groups showed a stronger synchronisation of their activity rhythms, which was perturbed when they were housed in groups containing flies with a genetically ablated circadian clock. This key finding demonstrates that social cues modulate the timing of activity in a clock-dependent manner. Similar experiments have evaluated sleep levels of individual animals after long periods of social enrichment in developmentally mature adults (Ganguly-Fitzgerald et al., 2006). Socialisation in 1:1 male:female groups during 4 days showed a group-size-dependent increase in sleep that is dependent on the dopaminergic system (Ganguly-Fitzgerald et al., 2006). Interestingly, the increase in sleep resulting from socialisation is restricted to daytime sleep. This observation indicates that day and night sleep may be differentially regulated. Alternatively, increased sleep during the first part of the day could mean sleep pressure has already dissipated by night-time. Importantly, similar results were shown for groups of females housed in groups of 30 for 9 days (Zimmerman et al., 2012). Moreover, this increase in sleep after social enrichment is dependent on the flies’ genetic background, specifically the presence of the Rover variant of the foraging gene (Donlea et al., 2012).

Actually, the sleep increase observed after socialisation may be necessary to downscale synapses and restore branch length, branch points, and spine number to basal levels^6^ (Bushey et al., 2011). These experiments, together with a plethora of other results collected in different species, support the synaptic homeostasis hypothesis: wakeful experience results in potentiation, some of it is useful and some redundant. During sleep, synapses are downscaled returning brain activity to basal levels. This pruning process allows further potentiation during subsequent wakefulness (Tononi and Cirelli, 2006).

It is clear that social interaction during adulthood regulates sleep. Likewise, this is true for social interactions occurring during critical periods of development, both during the larval stage and the first days of adult life. In particular, high larval density causes greater sleep consolidation during adulthood, a phenotype that is clearer in females than in males (Chi et al., 2014). Similarly, young adult flies exposed to a social environment present higher levels of sleep, which is reversible if the flies are kept in isolation. This process is dependent on the core clock gene *per* (Donlea et al., 2009), but it is independent of other core clock genes like *timeless*, *cycle*, and *clock* (Ganguly-Fitzgerald et al., 2006). This may indicate that this is a clock-independent process in which *per* has a separate function.

It is important to highlight that these reports measure sleep in isolated flies following exposure to a social environment, but not during the social interaction itself. Altogether, these data allow clear conclusions relating to the regulation of sleep by social experience, which can be interpreted as a homeostatic response to sleep loss during the interaction. However, the ongoing interactions throughout a group are critical because the behaviour of individual members is not a good predictor of the group-level activity (Higgins et al., 2005). Thus, the question remains: how do flies sleep *during* the social interaction? In an attempt to evaluate the sleep of populations of flies, Liu et al. (2015) studied the overall activity of mixed and same-sex populations. Their main conclusion is that same-sex groups coordinate their sleep, showing a temporal pattern similar to that of an individual. However, population records show lower levels of sleep compared to an isolated fly, which is expected since this system would only record sleep when all the flies sleep in unison. In agreement with previously described data, mixed populations exhibit lower sleep than populations of female flies alone, which may be explained by the fact that males exhibit higher activity levels due to sexual arousal. Finally, sleep-deprived populations exhibit homeostatic regulation characterised by a rebound sleep. Thus, beyond the lack of an individualised assessment of sleep during housing the authors conclude that socialisation modulates sleep amount but does not obliterate the two main regulators of sleep, the clock and the homeostat.

Beyond interesting results described above, we think that a description of sleep regulation during the presence of conspecifics is still missing. We believe that recently developed tools to track individual flies within populations (Kain et al., 2013; Perez-Escudero et al., 2014; Klibaite et al., 2017; Liu et al., 2018; Scaplen et al., 2019), when used in combination with existing open source tools to analyse sleep data (Geissmann et al., 2019b), will enable us to address this particular aspect of sleep regulation. We are of the opinion that a naturalistic approach to the subject of sleep regulation will ensure clearer, more meaningful conclusions, and technical developments will be fundamental to achieving these goals.

## Concluding Remarks

Sleep is critical but must remain a highly plastic behaviour to allow an organism to adapt to its ever-changing environment. Here we have discussed how three main environmental factors, temperature, food availability/quality, and social context can regulate sleep. Information about these ecological relevant contexts reaches the brain through the sensory systems, which act as an interface between the individual and its environment. In particular, an array of peripheral systems conveys information regarding ambient temperature, food availability, and presence of potential mates and aggressors/competitors. Importantly, these ecologically relevant cues are in large conveyed through chemoreception (Linford et al., 2012, 2015; Beckwith et al., 2017) and in the case of temperature, the family of temperature-regulated TRP cationic channels that transmit both innocuous and nociception information (Dillon et al., 2009). Based on the meaning conveyed by the signal itself, sensory perception is sufficient to regulate the quantity (Linford et al., 2015; Beckwith et al., 2017) and architecture of sleep (Linford et al., 2012; Hasegawa et al., 2017).

A key point is that sensory information serves to instruct behaviour in response to environmental change. For example, the absence of food-related cues or high temperature triggers food searching or escape behaviour, respectively. These behavioural modifications can also influence sleep in two main ways. Firstly, engaging more in a particular behaviour such as mating or foraging can ultimately result in less sleep simply through redistribution of a finite time budget. In addition to external cues, changes in the internal state can also drive this re-allocation of time to certain behaviours. For example, mated females engage more in egg laying, sexually aroused males relentlessly engage in courtship dismissing the need of sleep and starved flies engage in food searching as opposed to sleep. Secondly, external factors such as the presence of conspecifics of the same sex modulate the quality of wakeful experience, leading to increased need for sleep following the encounter (Bushey et al., 2011). However, following some wakeful experiences, like the mating rejection that males experiences after courting a mated female, sleep debt is not repaid *per se*, at least not in total amount of sleep. Interestingly, it is a possibility that the depth or intensity of sleep could work as a compensatory mechanism to dissipate sleep pressure. Thus, how sleep loss, as a result of different wakeful experience, determines future sleep need is not fully understood yet.

From a mechanistic perspective, external factors could differentially affect levels of oxidative stress. It is well documented, for example, that caloric restriction (Ungvari et al., 2008) and even social interaction (Ruan and Wu, 2008) can reduce accumulation of reactive oxygen species (ROS) whereas aggressive encounters may promote ROS production (Ramin et al., 2019). A recent and emerging concept in the *Drosophila* sleep field is that sleep is affected by levels of oxidative stress, and sleep deprivation can accelerate accumulation of ROS (Hill et al., 2018; Kempf et al., 2019). Then, it is plausible to hypothesise that mechanisms facilitating sleep loss or gain, both during or after environmental change, could be in part explained by the sensing of ROS levels. Thus far, this idea has been relatively unexplored but an avenue worth pursuing.

Sleep loss ultimately comes at a cost and understanding how animals weigh the cost and benefit of engaging in other behaviours instead of sleep and vice versa is a key biological quandary that begs to be investigated. A wide range of factors can influence sleep, yet these seemingly independent variables are in fact highly interactive. For instance, a manipulation such as mating can shift an animal’s entire behavioural profile (Geissmann et al., 2019a). After mating, females not only spend more time egg laying and less time sleeping, their nutritional requirements change. Thus, sleep loss phenotypes observed in mated females may be exacerbated by nutrient deficiency, which is also a major regulator of sleep. Similar examples exist in which the complexity of behavioural regulation and the limitations of our methodologies can contaminate our conclusions. For instance, caffeine is thought to influence sleep through its psychostimulatory effects on the brain; however, this phenotype is likely confounded by taste-driven changes in food intake. Equally, *Drosophila* neurogenetics sometimes encompass the use of thermogenetics to manipulate neural circuits governing behaviour. As discussed, temperature has drastic effects on sleep, even in basal conditions, making data interpretation more intricate.

From a personal perspective, we believe that it is crucial to embrace the complexity and interactivity of behaviours to improve the output of our science. We encourage the community to use tools that describe behaviour more accurately, build bridges between seemingly independent fields of research and try to agree upon standards, such as rearing environmental conditions and diets, in order to make research more coherent, facilitating the reproducibility of data and the comparison of results.

**FIGURE 4:**
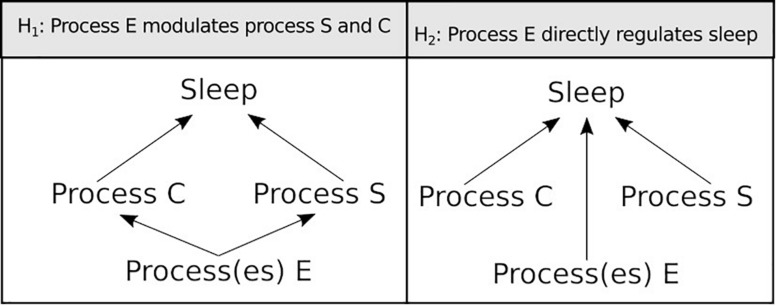
Two alternative hypotheses on how environmental factors can be integrated into the Borbély (1982) model of sleep regulation.

Finally, the prevailing idea is that the timing and quantity of sleep is controlled by two main processes, the circadian clock and the sleep homeostat. However, while this model has provided an important framework for understanding sleep, we now must try to understand and model how external and internal factors can perturb or interfere with sleep regulation. We see two main ways in which to incorporate environmental factors into the model of sleep regulation. One interpretation is that all the factors that affect sleep are modulators of the sleep homeostat. Under this scenario the two-process model remains intact but incorporates several layers of regulation within the S process and perhaps Process C (Figure 4, left panel). Alternatively, external factors composing a third process (Process E), or even several new processes (as many as factors can be identified) can be added to the model as direct regulators of sleep. This latter interpretation would have a corollary: process C informs the timing of sleep; process S encodes the need to sleep based purely on the tiredness; and Process E will antagonise or synergise with the need to sleep based on perceived weight of competing behavioural drives (e.g., need to eat or mate) (Figure 4, right panel). Importantly all these processes should convey the information to a centralised sleep arm that ultimately triggers the behaviour. Beyond this latter and favoured explanation, the fact that temperature, food availability, and social experience can regulate sleep suggests a high level of plasticity: sleep is context dependent and relative to many of the needs of the individual.

## Author Contributions

Both authors listed have made a substantial, direct and equal intellectual contribution to the work, and approved it for publication.

## Conflict of Interest Statement

The authors declare that the research was conducted in the absence of any commercial or financial relationships that could be construed as a potential conflict of interest.

## Footnotes

1 Most behavioural experiments to measure sleep or activity rhythms are conducted at 25°C.

2 Temperature can change sharply in the spatial dimension when animals move from or to shadow/sunny areas, this signals need to be ignored by the system controlling the rest–activity cycles.

3 It should be noted that lower concentrations of sorbitol reduce the total amount of sleep, thus this phenotype is concentration dependent.

4 Tsao et al. (2018) screened 34 MBON lines for impairments in food seeking behaviour under starved conditions. They then investigated the response of six corresponding DAN lines to starvation-induced potentiation, which is not exhaustive. These were either not screened by Sitaraman et al. (2015b) for sleep phenotypes or did not have a sleep phenotype. Nor is it known whether starvation-induced potentiation is a characteristic of all DANs or specific to only a subset investigated by Tsao et al. (2018).

5 It should be noted that the two studies do not use the same GAL4 drivers to label PAM clusters.

6 Bushey et al. (2011) studied downscaling specifically in a group of visual neurons, so it is not known whether this is universally applicable.
